# Survivin Expression in Luminal Breast Cancer and Adjacent Normal Tissue for Immuno-Oncology Applications

**DOI:** 10.3390/ijms241411827

**Published:** 2023-07-23

**Authors:** Sharon Wright, Scott R. Burkholz, Cathy Zelinsky, Connor Wittman, Richard T. Carback, Paul E. Harris, Tikoes Blankenberg, Charles V. Herst, Reid M. Rubsamen

**Affiliations:** 1 Saint Mary’s Regional Medical Center, Reno, NV 89503, USA; swright@westernsurgical.com (S.W.); zelinskicath@yahoo.com (C.Z.); connorwittman@gmail.com (C.W.); 2 Western Surgical Group, Reno, NV 89502, USA; 3 Flow Pharma Inc., Warrensville Heights, OH 44128, USA; sburkholz@flowpharma.com (S.R.B.); rcarback@flowpharma.com (R.T.C.); peh1@columbia.edu (P.E.H.); teakb@aol.com (T.B.); cvherst@flowpharma.com (C.V.H.); 4 Shasta Pathology Associates, Redding, CA 96001, USA; 5 Cleveland Medical Center, University Hospitals, Cleveland, OH 44106, USA; 6 Case Western Reserve School of Medicine, Cleveland, OH 44106, USA

**Keywords:** breast cancer, immuno-oncology, bioinformatics, artificial intelligence, next-generation sequencing, survivin, BIRC5, tumor associated antigen

## Abstract

Survivin (BIRC5) is a tumor-associated antigen (TAA) overexpressed in various tumors but present at low to undetectable levels in normal tissue. Survivin is known to have a high expression in breast cancer (e.g., Ductal Carcinoma in situ (DCIS) and triple negative breast cancer). Previous studies have not compared survivin expression levels in DCIS tumor samples to levels in adjacent, normal breast tissue from the same patient. To ensure the effective use of survivin as a target for T cell immunotherapy of breast cancer, it is essential to ascertain the varying levels of survivin expression between DCIS tumor tissue samples and the adjacent normal breast tissue taken from the same patient simultaneously. Next-generation sequencing of RNA (RNA-seq) in normal breast tissue and tumor breast tissue from five women presenting with DCIS for lumpectomy was used to identify sequence variation and expression levels of survivin. The identity of both tumor and adjacent normal tissue samples were corroborated by histopathology. Survivin was overexpressed in human breast tissue tumor samples relative to the corresponding adjacent human normal breast tissue. Wild-type survivin transcripts were the predominant species identified in all tumor tissue sequenced. This study demonstrates upregulated expression of wild type survivin in DCIS tumor tissue versus normal breast tissue taken from the same patient at the same time, and provides evidence that developing selective cytotoxic T lymphocyte (CTL) immunotherapy for DCIS targeting survivin warrants further study.

## 1. Introduction

Surgical procedures for the removal of breast cancer tumors are commonly combined with pharmaceutical or radiation therapies to kill the remaining tumor cells in the adjacent tissue and/or metastases [1]. While surgery is generally effective in increasing survival in some patients, chemotherapy or radiation therapy can result in “off-target collateral damage to normal cells and is associated with a wide range of treatment-associated morbidity [2]. Targeted approaches for adjuvant therapy, such as monoclonal antibody treatment of human epidermal growth factor receptor 2 (HER2)-expressing breast cancer tumors or treatment with checkpoint inhibitors such as anti-PD-L1 (Programmed Death Ligand 1) and PD-1 (Programmed Death Protein 1), have been shown to be effective in slowing down or preventing tumor recurrence but are also associated with treatment-related side effects [3,4,5].

Immunotherapy designed to expand cytotoxic T lymphocytes (CTLs) that recognize Major Histocompatibility Class I molecule (MHCI)-presented tumor antigens on tumor cells, including survivin, have shown encouraging results in small clinical trials [6,7,8,9]. Survivin is highly expressed during embryonic development and within cancer cells but is largely absent in adult tissues. A notable exception to this classification of survivin as an oncofetal antigen is that it is expressed in some regenerating hepatocytes, chondrocytes, specific populations of stem cells, endothelial cells, thymus, and testes [10,11,12,13].

Normal tissues adjacent to tumors exhibit inflammation and alterations in the extracellular matrix, thereby influencing RNA and protein expression [14,15]. Examining the expression of tumor-associated antigens in tumor-adjacent tissues is important for developing immunotherapies with optimal clinical safety and efficacy. Survivin expression in paired breast tumors and adjacent normal tissues has not yet been studied. In this study, we analyzed survivin expression in breast tumor samples obtained from patients undergoing surgery for ductal carcinoma in situ (DCIS) using snap-frozen tissue samples. Because the expression of specific survivin isoforms in breast cancer tissue correlates with poorer outcomes [16,17] and survivin in non-tumor tissue (e.g., reactive normal breast tissue) may provoke unwanted autoimmune effects, we sought to characterize the complete expression profile of survivin in tumor and normal autologous tissues. Such studies may be valuable for predicting the possible “off-target” effects of survivin-based T cell immunotherapy [9].

## 2. Results

**Figure 1:**
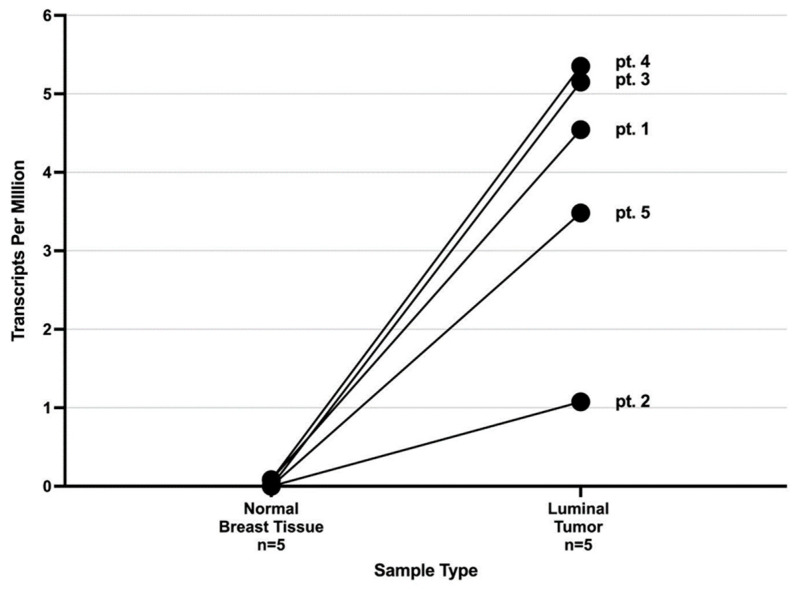
Expression level from mRNA-Seq of wild-type Survivin transcript (ENST00000350051) in normal and tumor breast tissue from the five subjects described in this study.

**Figure 2:**
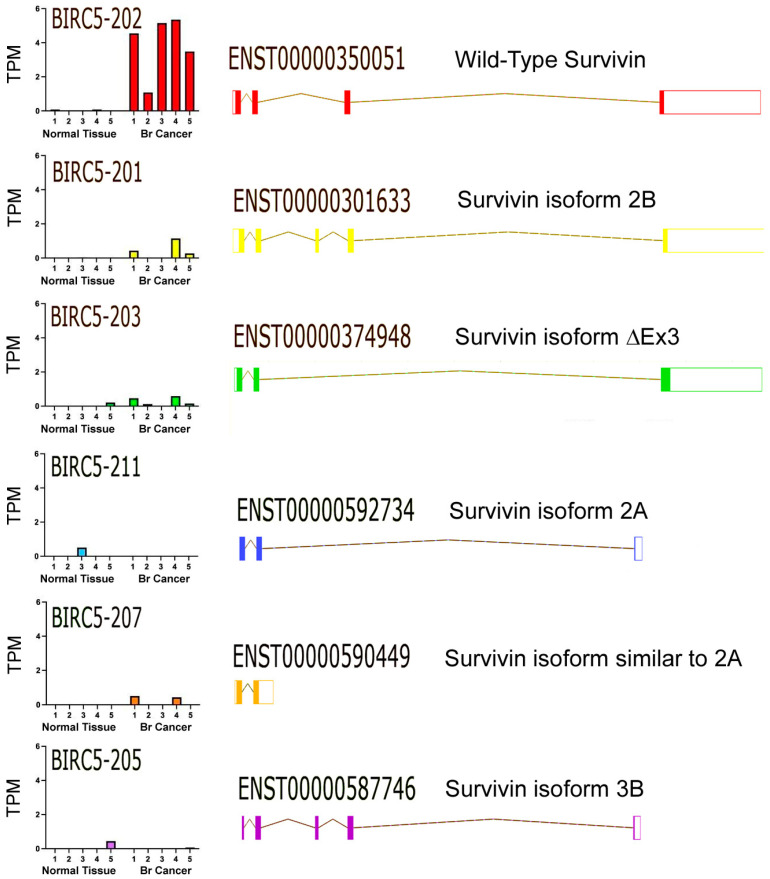
Survivin isoform expression in a cohort of five ductal cell carcinoma in situ (DCIS) patients. Survivin mRNA isoform expression was measured in five paired samples of DCIS breast tumor tissue and adjacent normal breast tissue by RNA-seq. The left panels show the abundance (as transcripts per million (TPM)) of each survivin isoform found in each normal breast tissue sample or paired DCIS tumor sample. The right-hand panels show the intron (colored line)-exon (colored box) structure for each survivin isoform detected. The biological significance of the expression of the different isoforms is discussed in the accompanying text.

All patients in this study had luminal breast cancer tumors, characterized as estrogen receptor-positive, progesterone receptor-positive, human epidermal growth factor receptor 2 (HER2)-negative, and low levels of KI-67 expression [18]. Survivin mRNA expression levels were measured in transcripts per million (TPM) of sequenced RNA samples. Survivin was found to be expressed in all five patient tumor samples in this study with the wild-type transcript, ENST00000350051, detected as the predominant form and the alternate splicing isoforms to a lesser degree [19] (Figure 1 and Figure 2).

The survivin transcript levels were higher than those observed in the respective pair-matched normal tissue samples. Given that typical breast cancer cells contain approximately 3–5 × 10^5^ mRNA molecules per cell [20], a TPM level above 1 suggests that a particular transcript is functionally expressed in the breast cancer cell population. Survivin expression in matched normal breast tissue samples was at or near 0 TPM, suggesting a limited background transcription. Gene expression in samples is often described relative to the total number of RNA transcripts in the sample (i.e., TPM). This can be confounding when characterizing tumor-associated proteins because it is not always straightforward to estimate sample purity, which is defined as the ratio of tumor cells to normal cells in a given tissue sample [21]. This has been documented for biomarkers in diagnostic kits, such as MammaPrint and OncotypeDX [22]. Table 1 shows the purity values of the tumor samples in our study, based on the determination of the aggregate areas of atypical epithelial hyperplasia or benign cells, as determined from stained slide images using automatic planimetry supervised by a pathologist.

Tissue sections for pathological analysis of tumor purity were obtained as close as possible to the sample used for sequencing for optimal comparison. These purity calculations were repeated four times before averaging, with all repetitions producing values within 10% of one another. Two of the five patient samples were less than 90% pure; however, these samples still expressed survivin at TPM levels significantly higher than normal tissue without correction based on purity. Further immunohistochemical results are provided in Appendix A.

**Figure 3:**
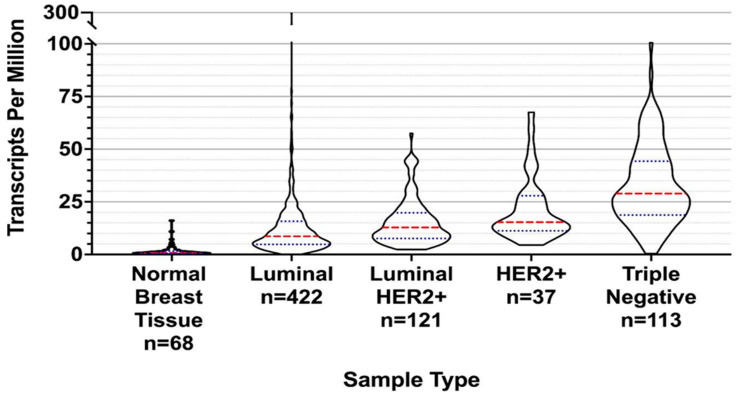
Violin plots outlining the expression of breast cancer samples from The Cancer Genome Atlas Breast Invasive Carcinoma (TCGA-BRCA) NIH/NCI database in terms of transcripts per method. The raw data from Tatlow et al. [23]. was processed for transcript per million quantification with the results plotted here. The overall width denotes the number of subjects with the corresponding transcripts per million frequency value shown along the Y axis within the indicated breast cancer subtype. The red lines represent median values, and the blue lines define quartiles.

The NIH Cancer Genome Atlas Breast Invasive Carcinoma (TCGA-BRCA) database contains nearly 1100 patients with different forms of breast cancer over the past 10 years (https://www.cancer.gov/tcga, accessed on 1 January 2022). The database creators categorized samples by cancer type, as determined by the immunohistochemical (IHC) assay [18]. As illustrated in Figure 3, all classifications of breast cancer expressed survivin at different levels. Triple-negative breast cancer survivin expression values were higher, on average, than those of other breast cancer types.

**Figure 4:**
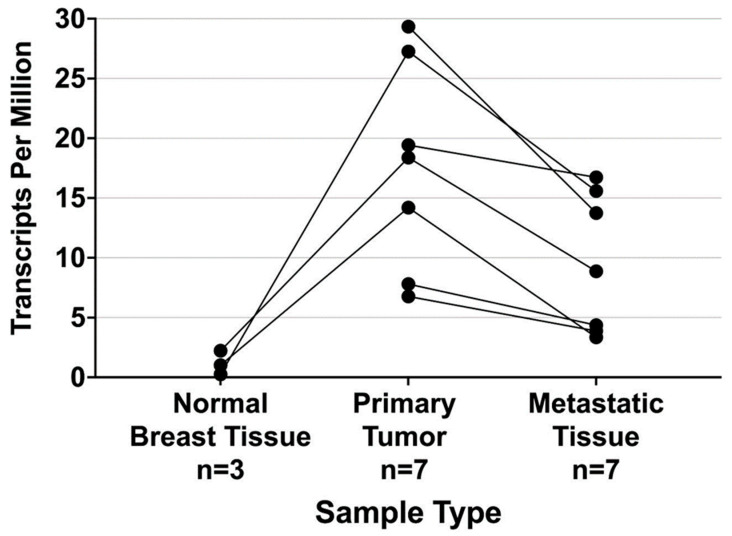
Wild type Survivin transcript (ENST00000350051) survivin expression levels measured by mRNA-Seq of autologous normal, tumor, and metastatic tissue samples obtained from breast cancer patients using the methods described by Tatlow et al. [23]. This plot was prepared using patient data cataloged in The Cancer Genome Atlas.

This may make survivin a particularly promising target for triple-negative breast cancer, which has few therapeutic options available to significantly improve outcome [24]. Within the TCGA-BRCA database, the wild-type transcript for survivin was also found in the mRNA samples obtained from metastatic tissues. We aggregated [23] sequence data from these samples into a database of 761 patients. The expression levels of survivin in metastatic tissue samples were lower than those reported in primary tumor tissue samples from the same individual (Figure 4). The presence of survivin in metastatic breast cancer tissue suggests that immunotherapy targeting survivin could be a useful part of breast cancer treatment, and that survivin targeting could be used for neoadjuvant therapy.

## 3. Discussion

For most cancer studies reporting RNA-seq expression levels, data from normal tissues adjacent to the tumor are unavailable, requiring databases to provide control RNA-seq levels for differential expression analysis. Although these databases can be valuable for exploring gene expression, the samples were not adjacent to the tumor and were not taken at the same time as the tumor excision. Using gene expression databases as a source of normal expression level controls raises concerns that the samples used could be degraded after formalin-fixed paraffin embedding (FFPE), as well as long ischemia times in deceased donors [25,26].

In this study, we focused on measuring survivin isoform transcript levels in DCIS. The biological and diagnostic significance of survivin and its various splicing variants has been previously reviewed [16]. Briefly, the most prevalent survivin isoform detected in the DCIS tissue analyzed in this study was the wild-type survivin isoform (BIRC5-202). This is the canonical form of survivin and is the most extensively studied isoform found in breast cancer [27,28]. The anti-apoptotic activity of survivin primarily rests in an baculovirus inhibitor of the apoptosis repeat (BIR) domain, thought to bind to cell death, thus promoting caspases directly and/or stabilizing other caspase inhibitors [29]. Survivin is primarily located in the nucleus and cytoplasm of dividing cells and is involved in regulating cell division, specifically the formation of the mitotic spindle and ensuring proper chromosome segregation. High levels of this survivin isoform are observed in many breast tumors, where it promotes tumor cell survival and contributes to chemo resistance [28,30].

The survivin 2B isoform, detected at background transcription levels in this cohort of DCIS samples, is also of general interest. The 2B isoform was reported to have reduced anti-apoptotic activity, possibly due to a disruption of the full length BIR domain and enhance sensitivity to chemotherapy, at least in leukemia [31]. Other survivin isoforms with truncated BIR domains include the survivin ΔEx3 and 2A isoforms [16], which were also detected at background in this study.

Epidermal growth factor receptor (EGFR), HER2, progesterone receptor (PR), Ki-67, PD-L1 and survivin are important gene markers associated with breast cancer. RNA-seq has higher sensitivity than immunohistochemistry (IHC) assays while also providing a higher throughput evaluation of RNA expression in the whole transcriptome. IHC is the most common assay used in clinical settings to detect breast cancer markers. Previous research has shown that there is a correlation between IHC and RNA-seq data [32], and current bioinformatics workflows reduce the potential for RNA-seq interpretation bias, suggesting that RNA-seq could be deployed in the clinical-diagnostic laboratory. Quantitative reverse transcription PCR (RT-qPCR) is also a valuable tool for cancer biomarker detection. However, similar to IHC, it is limited to a pre-designed set of genes for detection. Recently a number of novel methods for detecting survivin proteins mRNAs in complex mixtures (e.g., cell lysates) were reported (reviewed in [33]); these may also have clinical applications. These novel methods apply fluorescent or electrochemical reporters to antibody or nucleic acid-based detection methods to obtain lower limits of detections in the pM to nM range. RNA-seq may still have an overall advantage in that it allows for the quantitative analysis of both known and novel transcripts or isoforms, which is of particular interest for survivin, for which multiple isoforms have been described. In our sampling of DCIS tumor tissue, survivin was predominantly expressed as the wild-type transcript. However, five additional transcripts may encode proteins that could be potential targets for CTL attack. While the expression of these alternate transcripts has been observed at a lower level (Figure 2), these transcripts show an overlap between exons 1 and 2, which allows for consensus targeting using possible CTL epitopes contained in this region.

Limitations of this study include the limited sampling of normal tissue from the surgical site, the relative insensitivity of the RNA-seq methods applied in this study, and the potential issue that the tumor tissue was studied in bulk. More recently developed RNA-Seq methods, such as spatial single-cell or subcellular RNA-Seq, will allow for a higher resolution understanding of survivin expression within tumor tissue [34].

Various immunotherapies targeting the tumor-associated antigen survivin (BIRC5) have been described over the past two decades [35]. Clinical trials of immunotherapy targeting survivin have been performed in multiple cancer types, including melanoma, ovarian cancer, glioblastoma and prostate cancer [7,36,37,38,39,40,41,42]. Clinical trials of immunotherapies targeting survivin have generally been described as safe. Published approaches have focused on peptide-based drug delivery systems with survivin targeted sequences evaluated in vivo or predicted to bind to a particular human leucocyte antigen MHCI (HLA) type using various in silico techniques [6,13,37,43,44,45,46,47,48,49]. A wide range of delivery methods and adjuvants have been described in studies that have attempted to achieve an immune response that extends progression free survival. Despite the potential of survivin as a target for CTL attack in breast cancer patients, pivotal studies leading to the approval of survivin-based immunotherapy for breast cancer have not yet been conducted. Additional studies on peptide-based delivery systems coupled with techniques for the optimal selection of immunogenic HLA-restricted survivin protein peptide epitopes are needed to optimize immunotherapies targeting survivin.

Recent advancements have made immunotherapy a useful tool in the treatment of various cancers. However, the inability of checkpoint inhibitors to show efficacy against tumors not expressing PD-L1 [50] has limited their efficacy in the setting of breast cancer; for example, up to 70% of women with triple-negative breast cancer have tumors expressing low levels of PD-L1 [51]. TAA’s, such as survivin, provide the opportunity to develop immunotherapy, targeting a fixed set of peptides that could be broadly administered to breast cancer patients without the need for patient-specific tumor gene sequencing and personalized immunotherapy. Our recent preclinical studies in a mouse orthotopic 4T1 mammary tumor model have shown that microsphere vaccination with immunogenic MHC restricted survivin peptide epitopes and adjuvants significantly reduces tumor take and tumor growths rates [52], and support the concept of this generalized approach.

This study, describing the analysis of snap-frozen samples of tumor and normal breast tissue for gene expression of the survivin protein, showed that five out of the five patients studied had luminal breast cancer tumors secreting survivin at significantly higher levels than adjacent normal tissue taken from the excision site. This suggests that immunotherapy targeting breast cancer may be possible without destroying the normal breast tissue. Further work is required to develop a safe and effective immunotherapy targeting survivin protein TAAs, containing a suite of immunogenic HLA-restricted peptides across a broad population of patients.

## 4. Materials and Methods

This study was designed as a prospective observational investigation at a tertiary care center in a university hospital. The Institutional Review Board (IRB) of the University of Nevada, Reno, approved the study (Protocol ID: FP-BRST-2019 and Date of Approval: 3 August 2019), and the study was conducted in accordance with the Declaration of Helsinki. Inclusion criteria included the following; female subjects, 18 to 95 years of age having a planned surgical procedure for diagnosis or excisional treatment of all types of breast cancer, and able to provide consent or have a designated individual able to do so. A subgroup of these patients (*n* = 5) were enrolled in the study based on planned partial mastectomy as a treatment for Ductal Carcinoma in situ. This choice was based on the need to collect both tumor tissue and adjacent normal tissue with the lowest chance of invasive tumor tissue contaminating the normal adjacent tissue. All patients provided informed consent to participate in this study, coincident with the planned surgical partial mastectomy for the removal of previously diagnosed ductal carcinoma.

### 4.1. Breast Tissue Sample Preparation

As ischemia time and temperature of tissue samples greatly impact messenger RNA quality, the length of time between tissue excision and cryopreservation is no longer than 30 min [26,53]. Sterile conditions were maintained throughout the tissue-collection period. After tumor identification by palpation or radio-guided biopsy markers, the tumor was excised, marked for orientation, and sent for immediate pathological tissue handling for the study. A small piece of normal breast tissue was obtained (about 2 cm away from the tumor margin) from the surgical site, away from the tumor area, or from the medial aspect of the sentinel lymph node incision. Normal breast tissue samples were collected prior to tumor excision to minimize potential cross-contamination with the tumor tissue. Sealed e-beam-sterilized dissection kits were prepared and made available to the pathology staff for sample processing. The identities of tissues, either breast tumors or adjacent normal tissues, were confirmed by a pathologist using standard Hematoxylin and Eosin (H&E) staining of parallel frozen sections and formaldehyde-fixed, paraffin-embedded (FFPE) tissue blocks. FFPE blocks were examined using immunohistochemistry for HER, PR, estrogen receptor(ER), and Ki67 expression. The plane of the tumor tissue sample taken for gene sequencing was as close as possible to the plane sent for permanent blocks so that the H&E slide images would represent the sequenced region as closely as possible. Tissue that was not processed for pathology was dissected to produce approximately 100 mg pieces with dimensions less than 0.5 cm. These samples were snap-frozen in tubes capable of withstanding cryopreservation and labeled only with the patient’s unique code for that replicate. Tumor tissue samples were collected for DNA and RNA analyses, whereas normal breast tissue samples were collected for RNA analysis only. Peripheral blood mononuclear cells (PBMC) were harvested at a later date as the source of normal tissue DNA. An RNase inhibitor, RNAlater (Sigma Aldrich, St. Louis, MO, USA), was added to the samples for RNA sequencing immediately before snap freezing in liquid nitrogen to minimize RNA degradation during the thawing cycle at the next generation sequencing (NGS) laboratory.

### 4.2. Next Generation Sequencing

DNA and RNA were extracted from the respective samples using the QIAGEN reagent (QIAzol, QIAGEN, Hilden, Germany). Whole-exome sequencing was performed on the tumor and patient-matched blood PBMC. The input DNA for all samples was greater than 400 ng. Sequencing libraries were generated from the extracted DNA using the Agilent SureSelectXT2 Human All Exon V6 Kit (Agilent Technologies, Santa Clara, CA, USA). Normal and tumor tissue samples were sequenced at 100× and 300× coverage, respectively. Tumor tissue was sequenced at this depth to allow better characterization of variant allele frequencies and other structural variants (data not shown). RNA sequencing (RNA-Seq) was performed on the tumor and normal breast tissues to profile the transcriptome. The input RNA for all samples (tumor tissue and adjacent normal tissue) was greater than 400 ng. Messenger RNA was captured via poly A tails and prepared for sequencing using NEBNext Ultra II (New England Biolabs Inc., Ipswich, MA, USA). An Agilent 2100 bioanalyzer (Agilent, Santa Clara, CA, USA) was used to process samples, report the RNA integrity number (RIN), and visualize the ribosomal ratios. All RIN scores from the passing samples were above 6.0, with most samples scoring above 7.5. Normal samples were sequenced at 60 million paired-end reads and tumor samples were sequenced at 120 million paired-end reads. NGS was performed on all samples using NovaSeq6000 (Illumina, San Diego, CA, USA) with paired-end reads of 150 bp.

Human genomic data was uploaded to a secure Health Insurance Portability and Accountability Act (HIPPA)-compliant encrypted cloud storage system with redundant backup. All data were de-identified and organized using randomly generated patient study codes and sample types with associated hard-copy metadata and sample logs maintained in a fireproof locked safe.

### 4.3. Data Analysis

Genomic files were trimmed using FASTP sequences containing 20 adapters. Low-quality reads and those that were short or contained artifacts were removed during this process. Quality control was performed before and after the read trimming. Sequences were aligned to the GENCODE transcriptome using Kallisto, which pseudo-aligns the reads of individual transcripts per million. The Cancer Genome Atlas Breast Cancer Database Kallisto result files were downloaded and used for comparison [23,54,55,56]. The RNA-seq data described in this manuscript and database were run on version 0.43.0 of Kallisto and mapped to the GENCODE reference transcriptome, version 24. All sample transcript counts, study patients, and databases were joined and formatted using Python script [57]. Library size factor normalization and correction [23,58,59] was applied using Sleuth, version 0.30.0, running in R to allow for better sample-to-sample comparisons [60]. Survivin transcript values for tumor and paired normal tissues were extracted, their descriptive statistics calculated and plotted using GraphPad Prism, version 9 [61]. Based on a sample size of five paired samples and mean difference in TPM of Normal Tissue versus DCIS of 3.816, an SD of 1.8, we calculated the power of the study to be 0.83.

## Figures and Tables

**Table 1:** Proportions of tumor and normal cells in breast tumor samples used for RNA-seq.

Patient	Histopathology H&E Slide Interpretation ^1^	
	Malignant	Benign
1	50%	50%
2	97%	3%
3	95%	5%
4	95%	5%
5	65%	35%

^1^ The percentage of tumor (malignant) and healthy (benign) cells in tumor samples from patients in this study calculated from histological slides on the same plane as breast tissue tumor samples sent for RNA-seq.

## Data Availability

The code written in Python and R for the purposes described in the Methods section are available upon request.

## Appendix A

**Table A1:** Immunohistochemical Characteristics of Patient DCIS Tumor Tissue.

Patient	Estrogen Receptor (ER)	% ER Positive	ER Staining Intensity	Progesterone Receptor (PR)	% PR Positive	PR Staining Intensity	% HER2 Positive	KI67 Labelling Index < 10%
1	+	>60	Intermediate	−	0	N/A	0	✓
2	+	>99	Strong	+	>99	Strong	0	✓
3	+	>90	Strong	+	>90	Strong	0	✓
4	+	>90	Strong	+	>90	Strong	0	✓
5	+	>90	Strong	+	>80	Intermediate	0	✓
